# Impact of Bariatric Surgery in the Short and Long Term: A Need for Time-Dependent Dosing of Drugs

**DOI:** 10.1007/s11695-023-06770-5

**Published:** 2023-08-18

**Authors:** Cedric Lau, Charlotte van Kesteren, Robert Smeenk, Alwin Huitema, Catherijne A. J. Knibbe

**Affiliations:** 1 Department of Clinical Pharmacy, Albert Schweitzer Hospital, Albert Schweitzerplaats 25, 3318 AT Dordrecht, the Netherlands; 2 Department of Pharmacy and Pharmacology, Antoni Van Leeuwenhoek Hospital/The Netherlands Cancer Institute, Plesmanlaan 121, 1066 CX Amsterdam, the Netherlands; 3 Department of Clinical Pharmacy, University Medical Center Utrecht, Utrecht University, Heidelberglaan 100, 3584 CX Utrecht, the Netherlands; 4 Department of Surgery, Albert Schweitzer Hospital, Albert Schweitzerplaats 25, 3318 AT Dordrecht, the Netherlands; 5 Department of Pharmacology, Princess Máxima Center for Pediatric Oncology, Heidelberglaan 25, 3584 CS Utrecht, the Netherlands; 6 Department of Clinical Pharmacy, Sint Antonius Hospital, Nieuwegein & Utrecht, Koekoekslaan 1, 3435 CM Nieuwegein, the Netherlands; 7 Division of Systems Pharmacology and Pharmacy, Leiden Academic Centre for Drug Research, Leiden University, Wassenaarseweg 76, 2333 AL Leiden, The Netherlands

**Keywords:** Bariatric surgery, Roux-en-Y gastric bypass, Sleeve gastrectomy, Pharmacokinetics

## Abstract

Sparse information is available on pharmacokinetic changes of drugs over time after bariatric surgery. By reviewing the literature on the short- and long-term pharmacokinetic changes of drugs, several patterns were identified for 39 drugs. No relevant pharmacokinetic changes were identified for roughly a third of the drugs. Of the remaining drugs, levels were variable and partly unpredictable shortly after the surgery. In the long term, most of the drug levels remain altered, but in some cases they returned to preoperative values. Based on the changes and the efficacy-safety balance of each drug, clinicians may need to perform additional clinical monitoring for specific drugs, including measuring drug levels. This review provides suggestions for clinicians and pharmacists for specific time-dependent drug dosing advice.

**Supplementary Information:**

The online version contains supplementary material available at 10.1007/s11695-023-06770-5.

## Introduction

With the increasing number of patients undergoing bariatric surgery, there is a growing awareness of potential problems that can occur after bariatric surgery. Nutrient insufficiencies due to malabsorption are a common and known complication [1]. Less knowledge is available on the possibly altered pharmacokinetics of drugs due to malabsorption and other physiological changes. This may have challenging consequences for drug dosing after bariatric surgery [2].

To date, some reports suggest that pharmacokinetic alterations after bariatric surgery are time-dependent. In 2012, Hamad et al*.* [3] demonstrated that the plasma concentrations of sertraline and citalopram were initially decreased 1 month after a Roux-en-Y gastric bypass (RYGB). These levels returned to preoperative values in most patients at 6 months, and they sometimes even exceeded preoperative values at 1 year after the surgery. Steele et al*.* [4] described apixaban levels to increase at 1 month after surgery, to return to preoperative levels at 6 months and subsequently to decrease after 1 year. Thus far, changes in pharmacokinetics after bariatric surgery have mainly been described in studies with relatively limited numbers of patients. Moreover, pharmacokinetics was often assessed at a specific single time point or at a random time point regardless of the time after surgery (e.g., 1 to 9 years). Thus, the available data do not allow to distinguish the differences between the short- and long-term pharmacokinetics after bariatric surgery.

Dosing advice after bariatric surgery is greatly needed. Therefore, the European Association for the Study of Obesity has provided general dosing advice [5]. In the Netherlands, more specific dosing advice for patients after bariatric surgery has been incorporated in the Dutch national drug database. However, these current recommendations for dosing after surgery do not consider the influence of time after surgery. Such information could help clinicians and pharmacists in the treatment and follow-up of patients with bariatric surgery.

The aim of this review was to summarize the current knowledge regarding time-dependent changes in pharmacokinetics of drugs after bariatric surgery and to provide recommendations for dosing. Before presenting the methods and results, some background information on the 2 main types of bariatric surgery with associated physiological changes is provided.

## Bariatric Surgical Procedures and Associated Physiological Changes

After bariatric surgery, several physiological changes take place in the *short term*, i.e., up to 1 month after surgery, and in the *long term*, i.e., more than 1 month after surgery. This review focuses on the 2 most performed bariatric surgical techniques, the Roux-en-Y gastric bypass (*RYGB)* and the *sleeve gastrectomy (SG) *or* gastric sleeve.* The latter is solely a restrictive procedure, in which around 75% of the stomach is excised [6]. By contrast, RYGB is a malabsorptive-restrictive procedure, in which a small gastric pouch of around 30 ml is anastomosed to the distal limb of a jejunotomy performed at the mid-jejunum conform a Roux-en-Y construction [6]. This review did not include studies on gastric banding or biliopancreatic diversion (BPD)/duodenal switch. These techniques have lost popularity due to ineffectiveness and band-related complications in the case of gastric banding and nutritional complications in the case of BPD/duodenal switch. In recent years, other bariatric surgery procedures have been developed, such as one-anastomosis gastric bypass (OAGB), single anastomosis stomach-ileal bypass (SASI) or single anastomosis duodeno ileal-bypass (SADI). While these newer techniques are beyond the scope of the present review, they are expected to show similar pharmacokinetic alterations as RYGB.

### Gastrointestinal Tract

After both RYGB and SG, the scintigraphy data showed a reduction of gastric emptying time, both with and without food [7]. This reduction was observed for solids, semi-solids and liquids, both in the short term and in the long term up to 2 years [7].

In SG patients, the pH in the gastric pouch depends on the time after surgery [8]. Within a day after SG, the gastric pH rapidly increased from 1.7 to 5.0 [9]. One year after SG, the acid exposure returned to preoperative values [8]. In contrast to SG patients, the pH in RYGB patients remains high both in the short and long term after surgery [10]. In these RYGB patients, the median pH of the entire oro-cecal segment is increased from 5.5 to 7.0 [11].

Compared to the preoperative period, total bile acid concentrations in patients have been reported to increase after RYGB in a time-dependent manner over 5 years follow-up [12]. This is reflected in the increased risk of gallstone formation (cholelithiasis) in this group [13]. The inlet of bile and pancreatic fluid is delayed in RYGB patients [14].

### Liver

Drug-metabolizing cytochrome P450 (CYP) enzymes have been quantified in liver and intestinal biopsies of patients with RYGB on the day of surgery [15, 16]. However, no systematic information is available on CYP enzymes from biopsies *after* bariatric surgery.

Based on MRI data, the liver volume was decreased by 20% 3 months after bariatric surgery [17]. Subsequently, the liver volume remained relatively constant at 6, 12 and 24 months postoperatively at a decrease of 17 to 27% compared to the preoperative status. At 3 months after bariatric surgery, cardiac output was reduced by 20% and remained stable at this value up to the end of follow-up, which was 1 year after surgery [18]. The liver blood flow remains a constant percentage relative to cardiac output across BMI values in patients with obesity before surgery [19]. Therefore, the liver flow can be assumed to be approximately 20% lower after bariatric surgery.

### Kidney

After bariatric surgery, renal function may change over time. The extent of change seems to be dependent on the initial renal function. In patients with preoperative hyperfiltration, the glomerular filtration rate (GFR), as determined by iohexol clearance, normalizes at 1 year after surgery [20]. However, in patients with pre-existent renal impairment, GFR seems to remain unchanged [21]. The GFR in patients with a normal kidney function remain similar on the short term and is reported to decrease at 1 year after RYGB [22].

The assessment of the renal function before and after bariatric surgery can be challenging when using a serum creatinine-based estimate of the renal function [20]. The reliability of renal function estimates is affected directly by weight loss because of the decrease in muscle mass. When using an *indexed* estimated GFR, i.e., normalized estimated GFR to a body surface area of 1.73 m^2^, this can lead to underestimation in individuals with obesity [20]. It has been suggested that it might be better to monitor the renal function after bariatric surgery with a method based on an exogenous marker such as iohexol [20, 23]. Alternatively, the estimation of renal function can be improved by de-indexing the estimated GFR in patients with class III obesity [24]. However, *de-indexed* estimated GFR may slightly overestimate the renal function in patients after bariatric surgery regardless of whether they are still obese [25].

In clinical practice, changes over time in renal function in bariatric patients could be blurred if renal function is estimated based on serum creatinine. The values after surgery may not be directly comparable with preoperative values. However, the debate on how to assess and interpret renal function after bariatric surgery is still ongoing.

## Methods

Systematic searches in PubMed and Embase were performed using the following MeSH terms: “bariatrics”, “gastric bypass”, “sleeve gastrectomy”, “pharmacokinetics” and “time”. Furthermore, studies were also included if they contained the non-MeSH terms “drug concentration” or “drug level” and one of the following terms: “time after surgery”, “change*”, “long-term” or “short-term”. The search strategy can be found in the appendix. Studies were excluded if they were written in other languages than English, Dutch, German or French or if they included pediatric patients.

We included results from studies on drugs of which the pharmacokinetics were assessed on at least 2 different occasions after bariatric surgery. Pertinent citations from retrieved articles were also included. If available, the reported postoperative drug levels were compared to preoperative levels. Generally, if the area under the concentration-time curve (AUC) or trough level was between 80 and 120% of the preoperative values, the difference was considered to be non-relevant. If no preoperative values were available, the reported postoperative drug levels were compared to those in patients with obesity. Results on drugs for which data of less than 5 patients in total were available were described separately.

## Results

**Fig. 1:**
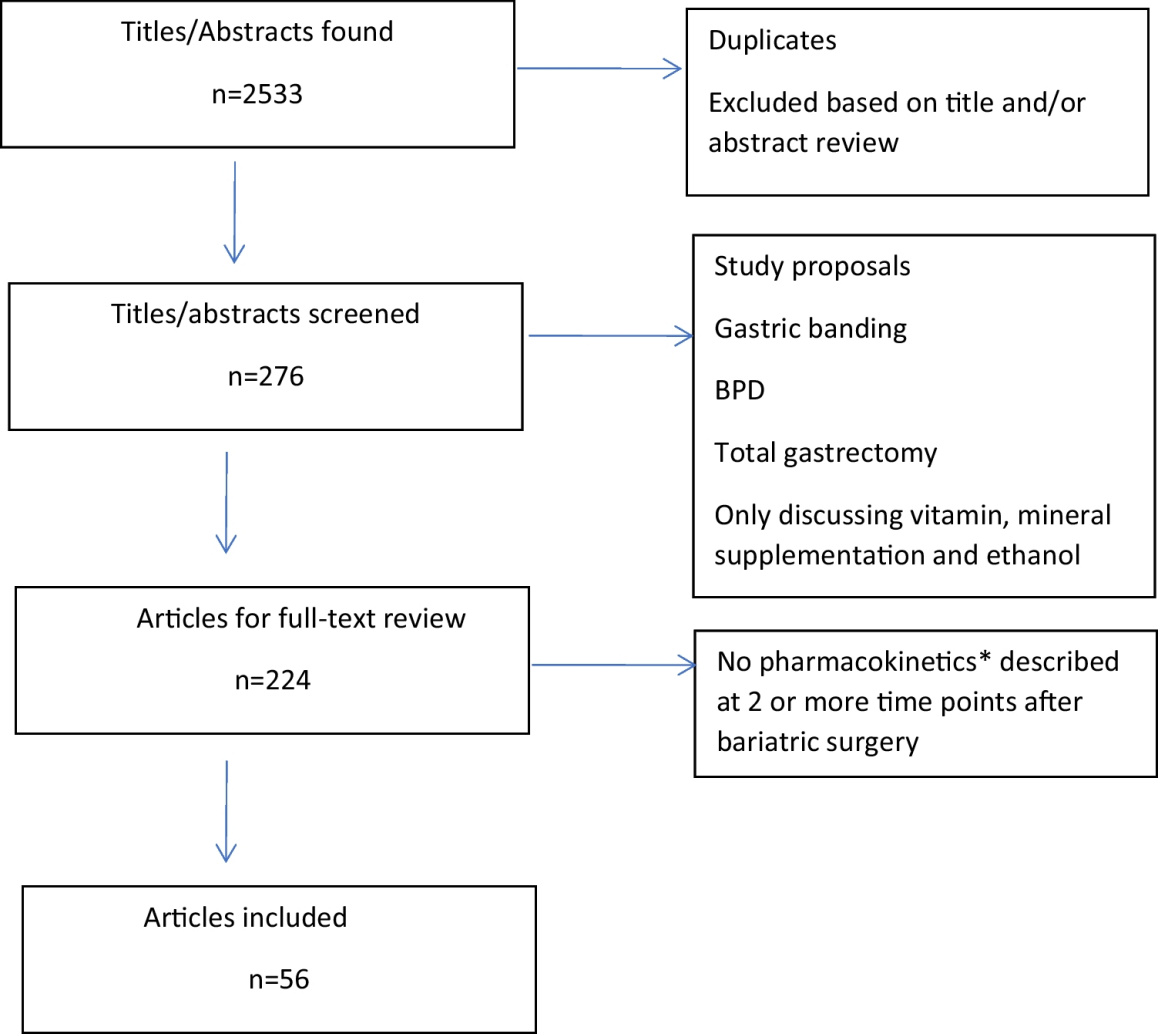
Flowchart of included articles. * For drugs whose pharmacokinetics are usually not measured, the used dosages and/or laboratory values were used (i.e., vitamin K antagonists and thyroid hormones). *BPD* = biliopancreatic diversion

**Fig. 2:**
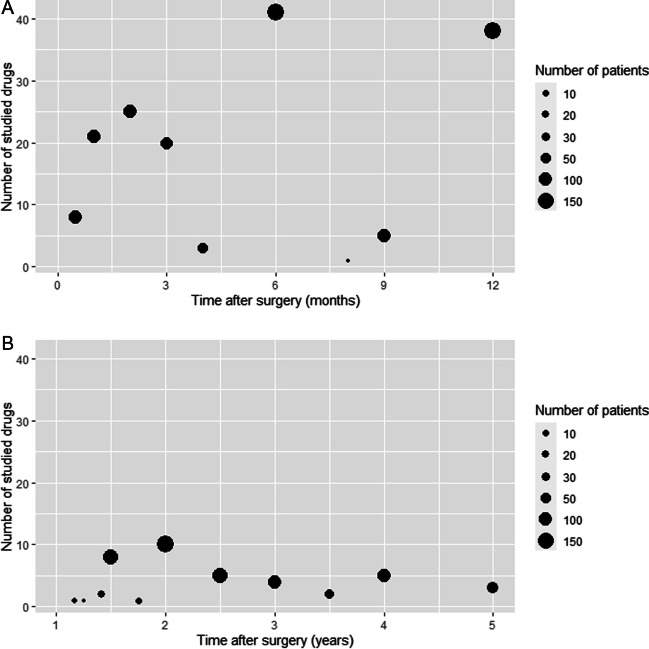
Numbers of studied drugs of which the pharmacokinetics were assessed at multiple time points up to 1 year after bariatric surgery (**A**) and from 1 to 5 years after surgery (**B**). The Y-axis depicts the number of studied drugs. The size of the dots indicates the total number of patients that were studied

Of the 2533 retrieved results, 276 articles were selected for screening after removing duplicates and reading title and abstract. After exclusion of study proposals, cases of gastric banding, BPD and total gastrectomy only, and studies discussing vitamin and mineral suppletion and ethanol, 224 results were reviewed (Fig. 1). In total, 56 articles described the pharmacokinetics of drugs at 2 or more time points after surgery. Of these 56 included articles, 22 investigated the effects of bariatric surgery on pharmacokinetics of drugs up to 6 months after bariatric surgery (Fig. 2a). The numbers of drugs studied beyond 1 year after bariatric surgery are shown in Fig. 2b.

Time-dependent effects on pharmacokinetic changes in patients with bariatric surgery were reported for 39 different drugs. The findings of 32 drugs for which data of ≥ 5 patients in total were available, are summarized in Table 1. The drugs are sorted by therapeutic area. A practical advice for clinicians is provided. Table 1 shows results of drugs for which altered pharmacokinetic was reported, while Table 2 shows drugs for which no pharmacokinetic changes were found. Since no relevant differences in pharmacokinetics between RYGB and SG were identified, no separate practical advice was provided for either type of surgery.

**Table 1:** Overview of the results from articles describing drugs with changed pharmacokinetics over time after bariatric surgery. Drugs are sorted by therapeutic group.

Class	Drug	Type of surgery	*N*	Observation time points after surgery within the same patient	Changes in pharmacokinetics, effects or required dose	Remarks	References	Practical advice
Changed pharmacokinetics over time								
Psychotropic drugs	Lithium	SG	1	2, 3 and 6 months	5 cases reported increased levels above a toxic level, requiring 4 hospitalizations. In 3 cases, levels increased within 2.5 weeks after surgery or after start of lithium therapy. 3 patients restarted lithium at a dose of 25–67% of pre-surgery. In 2 patients, a switch to extended-release product in a lower daily dose resulted in therapeutic levels comparable to preoperative.	Results can be influenced by change in renal function or inadequate fluid intake.	[26]	Close monitoring with TDM preferably weekly in the first month after surgery. Consider prior reducing the dose with 30–70% and titrate further based on TDM. Consider switch to extended release. Do not change to liquid formulation without TDM.
			1	17 days			[27]	
			1	5 years (9 and 18 days after start lithium)			[28]	
			1	Not described			[29]	
		RYGB	1	12 and 15 days			[28]	
	SSRI / SNRI (citalopram, escitalopram, sertraline, mirtazapine, duloxetine)	RYGB, SG	41	8 weeks, 6 months, 12 months	Sertraline (*n*=11) and mirtazapine (*n*=5): - at 8 weeks: decrease in dose-adjusted concentrations in 15/17 patients. - at 12 months: 41–51% reduction in dose-adjusted concentrations. Dose-adjusted trough levels returned to pre-surgery values in half of the patients. Citalopram/escitalopram (*n*=17) and duloxetine (*n*=8): - at 2 months: decrease in dose-adjusted concentrations in more than half of the patients. - at 12 months: the dose-adjusted concentrations tended to decrease further by 19–35%. - for 2 citalopram patients, the dose was doubled at 1 year after RYGB to achieve a similar trough concentration compared to pre-surgery.	Only trough levels were measured.	[30]	Monitoring of clinical effects in first year after surgery. Perform TDM on indication.
	SSRI/SNRI (citalopram, escitalopram, sertraline, duloxetine)	RYGB	7	1, 6, 12 months	Citalopram/escitalopram (*n*=4) and sertraline (*n*=2): - at 1 month: decreased levels in all patients to an average of 54% of preoperative values. - at 6 and 12 months: the levels returned in 4/6 patients to preoperative values. Duloxetine (*n*=1): - at 1 month: no significant change in AUC_0-7_ of duloxetine. - at 6 and 12 months: the AUC_0-7_ decreased by 56% and 80%, respectively.		[3]	Monitoring of clinical effects in first year after surgery. Perform TDM on indication.
	Escitalopram	RYGB	4	2 and 6 weeks	- At 2 weeks: decreased dose-adjusted levels in all patients with an average of -27% (range -4% to -71%) - At 6 weeks: further decrease of dose-adjusted levels in all patients to an average of -40% relatively to preoperative values.	One patient used omeprazole (CYP2C19 inhibitor). The decline in levels was smallest for this user, which may be attributed to the interaction with omeprazole.	[31]	
	Midazolam	RYGB	41	6 weeks and 2 years	At 6 weeks: no change in Cl and F, increase of Cmax by 50%. At 2 years: normalization of Cmax to preoperative values. Faster absorption and F decreased by 40%. No change in Cl.	Low dose: 1 mg intravenously and 1.5 mg orally	[16]	Titrate up to effect. Monitoring is warranted due to higher and earlier peak concentrations after oral administration.
			20	1 year	Compared to preoperative values, the Cmax was increased with factor 1.5, while Tmax halved. F was similar, while Cl was increased with factor 1.7.	5 mg intravenously and 7.5 mg orally	[32]	
			18	≥ 1 year	Compared to matched controls, Tmax was reduced by a third. Similar F and Cl. The other pharmacokinetic parameters remained similar. When administered intravenously, AUC_0-6_ was reduced by 43% compared to matched controls.	Low dose: 0.01 mg/kg intravenously and 1 mg midazolam orally	[33]	
			9	3 and 12 months	At both time points, Tmax was shortened from 0.6 to 0.3 h compared to preoperative status. Cmax increased with factor 1.7. Trend towards lower Cl/F. Trend towards lower AUC_0-24_	Only oral dose of 2 mg	[34]	
			10	5-8 weeks and 25-30 weeks	At both time points, Cmax doubled in a non-significant way, while Tmax halved compared to preoperative status. AUC_0-24_ remained similar.	Only oral dose of 2 mg	[15]	
	Pregabalin, lamotrigine	RYGB, SG	10	8 weeks, 6 and 12 months	Pregabalin (*n*=6) and lamotrigine (*n*=4) - at 8 weeks: 8/10 patients had higher dose-corrected trough concentrations. - at 6 and 12 months: the dose-corrected trough concentrations returned to pre-surgery values in 5/8 patients. In 1 lamotrigine patient and 2 pregabalin patients, the concentrations tended to rise over time.	Only trough levels were measured.	[30]	In epilepsy patients, it is advisable to monitor levels shortly after bariatric surgery. For other indications (e.g., neuropathic pain), clinical monitoring of the effects and adverse events is advisable.
	Lamotrigine	RYGB, SG	1	11 weeks	The lamotrigine level decreased by 90%, leading to increased seizure frequency and dose increase of 33%	Only 1 post-bariatric trough level was measured.	[35]	
	Levetiracetam	RYGB, SG	5	2 weeks up till 32 weeks	The changes compared to preoperative status varied among the patients At 2–4 weeks: - in 1 patient, the concentration increased by 156%, afterwards a dose reduction of 23% was applied. - in 1 patient, the concentration decreased by 66% and was associated with increased seizure frequency. The dose was doubled afterwards. At 18–32 weeks: - In 3 patients, the concentration increased by 14–23% without any clinical and medication changes.	Only 1 post-bariatric trough level measured per patient.	[35]	
	Carbamazepine	RYGB, SG	4	9 weeks up till 1.5 years	Epilepsy patients with controlled-release products - in 2 patients, the concentration was 45–82% lower than preoperative values. For one patient, the dose was increased by 20%. - the concentration did not change in another patient, but due to nausea and vomiting a dose reduction of 11% was applied. - in 1 patient the concentration increased with 39% which was well tolerated.	Only 1 post-bariatric trough level was measured per patient.	[35]	In epilepsy patients, it is advisable to monitor carbamazepine levels shortly after bariatric surgery. For other indications (e.g., neuropathic pain), clinical monitoring of the effects and adverse events is advisable.
		SG	1	4 weeks, 5 weeks, 10 weeks	At 4 weeks: nausea, vomiting, lethargy and agranulocytosis. Trough level was toxic at 15.9 mg/L (ref: 4–12 mg/L). The carbamazepine dose was gradually reduced by 50%, after which carbamazepine levels dropped and remained between 8–10 mg/L in the subsequent weeks with resolution of the symptoms.	Only trough levels were measured. No preoperative value of carbamazepine level was provided.	[36]	
	Valproic acid	RYGB, SG	3	1 week up till 1 year	All patients had sustained release formulations. - In 2 patients the concentrations after surgery decreased by 9 to 13%, which was clinically not relevant. - In 1 patient the level was decreased by 65% after 1 year, with increased seizure frequency. Doubling of the dose was required.	Only 1 post-bariatric trough level was measured per patient.	[35]	In epilepsy patients, it is advisable to monitor valproic acid levels shortly after bariatric surgery. For other indications (e.g., bipolar disorder), clinical monitoring of the effects and adverse events is advisable.
		RYGB, SG	2	8 weeks, 6 and 12 months	No significant differences were found in dose-adjusted trough concentrations.	Only trough levels were measured.	[30]	
		RYGB	1	2 weeks, 5 weeks, 9 months, 1.5 year	A patient switched from extended release 1500 mg BD to immediate release 1000 mg TID just prior to RYGB. - 2 and 5 weeks: total and free concentration were within therapeutic range: 66-82 mg/L and 13–15 mg/L, respectively. - 9 months: the total and free levels dropped to 26 and 4 mg/L, upon which the dose was increased by 50%. - 10 months and 11 months: the total and free levels remained subtherapeutic. The dose was increased to double the amount of the preoperative dose. - 12 months and 1.5 years: the total and free levels were within therapeutic range (52–62 mg/L and 5 mg/L respectively.)		[37]	
	Phenytoin	RYGB, SG RYGB	3	1 up till 15 weeks 2 weeks, 5 weeks, 9 months, 1.5 year	In 2 patients, the phenytoin levels were 34% and 10% lower than pre-surgery. No medication changes were necessary. In 1 patient, the concentration increased by 7% compared to preoperative status. However, due to somnolence the dose was reduced by 15%.	Only 1 post-bariatric trough level was measured per patient. Phenytoin possesses non-linear kinetics.	[35]	In epilepsy patients, it is advisable to monitor phenytoin levels after bariatric surgery. Do not switch extended-release formulations to immediate formulations.
					A patient switched from phenytoin extended release 200 mg BD to phenytoin 200 mg BD (immediate release formulation) just prior to RYGB. - 2 weeks: total concentration decreased from 7 mg/L to 5.8 mg/L and free concentration decreased from 1.5 mg/L to 1.1 mg/L. The dose was increased by 20%. - 5 weeks and 9 months: the total and free concentrations returned within therapeutic range. - 1.5 years: the total and free concentrations increased to 15.7 and 3.1 mg/L, upon which the dose was returned to the original dose.	By converting phenytoin extended release to immediate release and keeping the same dosing frequency, trough levels are expected to decrease. Albumin values were not provided.	[37]	
RYGB			1	2 years	After being free from seizures for 30 years, a patient suffered from a seizure at 1 year after RYGB. Despite doubling the dose of phenobarbital to 60 mg BD and adding phenytoin 300 mg per day, a second seizure occurred 2 years after RYGB. Phenytoin levels were undetectable (< 3 mg/L). Dose of phenytoin was increased to 500 mg OD with no effect in measured phenytoin levels.	Medication adherence was not described No preoperative value of anti-epileptic values provided.	[38]	
Antibiotics	Amoxicillin	RYGB	8	2 months	AUC_0-8_ increased by 255% and Cmax by 185% compared to pre-surgery. No change in Tmax.	Single dose 500 mg capsule	[39]	No dose adjustment recommended.
		RYGB	20	3 months up till 10 years	At a longer follow-up of at least 3 months until 10 years, the AUC_0-inf_ was approximately 50% lower in RYGB patients receiving the suspension and tablets than healthy subjects receiving the capsules.	Only 1 post-bariatric occasion per patient. Results can be influenced by differences in formulations and dosages due to non-linear absorption of amoxicillin.	[40, 41]	
Cardiac drugs	Apixaban	RYGB SG	14 14	1, 6 and 12 months	At 1 month, AUC_0-72_ increased by 22%. T_1/2_ increased from 9.4h to 12.2 h. At 6 months, AUC_0-72_ and t_1/2_ returned to baseline levels. At 12 months, AUC_0-72_ decreased by 17%. T_1/2_ was similar to baseline level. Tmax and Cmax were not significantly different throughout the study period. In contrast to apixaban drug levels, the predose Xa activity changes were as follows: - at 1 month: decrease by 13%. - at 6 months: activity remained 14% lower than preoperatively - at 12 months: activity was 21% lower than preoperative values.	Single dose 5 mg Changes in Xa activity maybe due to weight loss and not only to differences in pharmaco-kinetics.	[4]	Consider switching to vitamin K antagonists, as those drugs can be monitored using INR.
	Atorvastatin	RYGB	12	3-8 weeks and 21-45 months	Large variability in atorvastatin concentrations with overall slightly increased median AUC_0-8_ exposure of 20%, which returned to pre-surgery values after 2 years. Tmax doubled over time, while there was large variability in Cmax.	Improvement of LDL could be due to RYGB itself.	[42]	No *a priori* dose adjustment indicated. Titrate based on LDL. Reconsider need of a statin at longer term
			3	3 and 6 months	Mean dose-corrected and weight-corrected concentrations of atorvastatin were decreased by 58% at 3 months and 75% by 6 months. A similar trend was observed for the active metabolites of atorvastatin.	Sampling of non-trough levels may not be reflective for total exposure. Weight-based concentrations unusual	[43]	
	Simvastatin	RYGB	5	3, 6 and 12 months	Large variability in the magnitudes and directions of the changes. Overall mean dose-corrected and weight-corrected simvastatin and metabolite concentrations increased by 33% and 150% respectively at 3 months and more than doubled at 6 months post-RYGB. The concentrations tended to decline to pre-operative levels at 1 year.	Sampling of non-trough levels may not be reflective for total exposure, especially because simvastatin is a prodrug and has a short half-life. Weight-based concentrations unusual	[43]	No *a priori* dose adjustment indicated. Titrate based on LDL. Reconsider need of a statin at longer term
	Rosuvastatin	RYGB	4	3 and 6 months	Mean dose-corrected and weight-corrected rosuvastatin levels dropped by 43% at 3 months and continued to drop further by 61% at 6 months compared to preoperatively.	Sampling of non-trough levels may not be reflective for total exposure. Weight-based concentrations unusual	[43]	No *a priori* dose adjustment indicated. Titrate based on LDL. Reconsider need of a statin at longer term
	Warfarin	RYGB	27	8-14 days, 3, 4, 5, 6, 7, 8 weeks, 3 and 6 months	The weekly dose of warfarin dropped with 7.7 mg at days 8–14 after surgery, 15.8 mg at week 3–5 and 30 mg at days 50–56. However, the weekly dose returned to pre-surgery doses at days 90–180.		[44]	Monitor INR more frequently in the first 6 months. Doses needed to reach an adequate INR may decrease shortly after SG and RYGB, and then return to pre-surgery doses.
		RYGB, SG	53	1, 6, 12 and 36 months	The average daily dose was decreased by 26% at 1 month, 18% at 3 months, 13% at 12 months and 7% at 36 months.		[45]	
	Digoxin	RYGB	9	3 months and 12 months	Tmax was halved at 1 year, while Cmax and AUC_0-24_ remained similar		[34]	No dose adjustment recommended
Metabolic drugs	Hydrocortisone	SG	1	3, 9 and 15 months	AUC_0-inf_ of hydrocortisone increased over time: +24% at 9 months after SG, and further to +74% at 15 months after SG. The required daily hydrocortisone dose decreased from 38.5 mg once daily (OD) to 35 mg OD at 3 months, to 25 mg OD at 9 months and 23 mg OD at 15 months after SG.	Cortisol concentrations were measured after intravenous doses of hydro-cortisone.	[46]	Titrate up or down to its effect. Measurements of cortisol levels could be helpful.
			1	2 weeks	Tmax of cortisol decreased with 33% and Cmax increased by 23%. The AUC_0-6_ increased by 24%.	Cortisol concentrations were measured after oral dose of hydro-cortisone.	[47]	
		RYGB	3	2 weeks	Tmax decreased by 27%. In a case series, 24 h cortisol profiles looked similar to pre-surgery. Median AUC_0-6_ and Cmax were unchanged.	Cortisol concentrations were measured after oral dose of hydro-cortisone.	[47]	
	Levothyroxine	RYGB SG	168	Follow-up in 5 original publications: from 1 week till 3 years. In 1 publication up to 9 years.	In 4/5 studies, patients needed a decreased levothyroxine dose, while in 1 study no changes in levothyroxine dose were reported. In 1 of 5 studies in RYGB patients, a higher levothyroxine dose was reported.	Differences between pharmaco-kinetics and pharmaco-dynamics/physiological changes cannot be distinguished based on the data from these studies.	Review article summarizing 5 studies [48] Original studies: [49–53]	Titrate dose based on thyroid function. Patients may need a lower dose over time
		RYGB SG	7 10	5 weeks	Levothyroxine AUC_0-4_, Cmax and Tmax remained similar in RYGB patients compared to pre-surgery values. AUC_0-4_ increased by 32% in SG patients compared to pre-surgery values, while Cmax and Tmax remained similar.		[54]	
		RYGB	15	2-3 months	Levothyroxine Cmax and AUC_0-300_ increased by 26 and 27% respectively compared to the control group. Tmax was not significantly altered.	Control group of pre-RYGB patients with hypothyroidism, who were not treated with thyroid hormones for 2 months.	[55]	
		SG	52	6, 12 and 24 months	The median daily dose of levothyroxine dropped in all patients, by 19% at 6 months, 13% at 12 months and 45% at 24 months. There was a significant positive correlation between weight loss and decrease in daily levothyroxine dose.	Differences between pharmaco-kinetics and pharmaco-dynamics/ physiological changes cannot be distinguished based on the data from these studies.	[56]	
		RYGB SG	27 6	6-48 months (every 6 months)	No significant changes in dosing occurred after 2 years after surgery. Until 2 years, the median defined daily dose dropped from 125 to 100 μg and returned to 125 μg.		[57]	
	Metformin	RYGB	16	17 months	After surgery, AUC_0-inf_ was 21% higher than in BMI-matched controls	Non-diabetic patients	[58]	Studies cannot be directly compared. No dose adjustment recommended. Titrate based on glucose control and need for antidiabetic therapy.
			24	3, 6 and 12 months	Ka and Vd were reported to be significantly reduced at 3, 6 and 12 months, but the exact changes were not clearly reported and cannot be adequately interpreted.	Only published as abstract	[59]	
	Omeprazole	RYGB	18	≥ 1 year	Tmax was reduced by 25%. The other pharmacokinetic parameters remained similar.		[33]	Faster absorption with initially higher Cmax, which seems to return to pre-surgery value over time. No dose adjustment recommended
			20	2 months	Omeprazole and metabolites concentrations measured at 90 min after administration were 30-50% lower than before RYGB.	Short sampling interval	[60]	
			34	≥ 6 weeks	The Tmax was shortened to less than half. The Cmax was 24% higher. AUC_0-12_ of omeprazole was reduced by 24%.		[61]	
			17	1 and 6 months	In RYGB patients, the weight-corrected Cmax was 38% lower at 1 month and 35% lower at 6 months after surgery. The weight-corrected AUC_0-12_ values were 52% and 45% lower at 1 and 6 months after surgery.	Weight-corrected Cmax and AUC unusual.	[62]	
			10	5-8 weeks and 25-30 weeks	Cmax increased by 96% at 5-8 weeks and then returned to almost pre-surgery values. Tmax was halved at both time points. AUC_0-8_ did not change statistically significantly.		[15]	
Analgesic drugs	Paracetamol	RYGB	10	1 and 6 months	Faster absorption and higher Cmax were reported. The Cmax increased by 30–38% at 1 and 6 months after surgery. The AUC_0-inf_ increased by 41–55% within 1-6 months after surgery.		[63]	Faster absorption and higher Cmax. No dose adjustment recommended.
			9	3 and 12 months	Cmax of paracetamol values were doubled at 3 and 12 months after surgery. Paracetamol AUC_0-inf_ increased by 1.6-fold and 1.5-fold respectively. Paracetamol-glucuronide and paracetamol-sulfate AUC_0-inf_ increased by 20% and 26% respectively at 3 months, but returned to pre-surgery values at 12 months after RYGB. Paracetamol-cysteine AUC_0-inf_ was not altered at 3 months post-RYGB and was reduced by 15% at 12 months after surgery. Paracetamol- Nacetylcysteine parameters were unchanged at all time points.	AUCs of paracetamol metabolites are the result of the AUC of paracetamol, the formation of the metabolite from paracetamol, the Vd and elimination Cl of the metabolite and should therefore be interpreted with caution.	[64]	
		SG	14	1 and 6 months	Cmax increased by 30-38% at 1 and 6 months after surgery. AUC_0-inf_ increased by 47% and 41% respectively. Tmax was halved at 6 months after surgery.		[63]	
	Morphine	RYGB	25	2 weeks and 6 months	After administration of immediate release formulation, the Tmax was 2-fold lower within 2 weeks and 7-fold lower at 6 months. AUC_0-12_ increased by 55% at 6 months. Cmax was 70% higher at 2 weeks and 230% higher at 6 months.		[65, 66]	Avoid immediate release formulations of morphine. If immediate release formulations are necessary, be aware of side effects and consider lower doses with a shorter interval or switching to another opioid.
			12	≥ 2 years	After administration of extended-release formulation, there were no differences in AUC_0-12_, Tmax, Cmax of morphine and its metabolites between RYGB patients and matched non-surgical controls.		[67]	
Oral contraceptives	Levonorgestrel	RYGB	15	1-9 years	The plasma concentrations did not differ from those of matched controls with BMI > 30 kg/m^2^. AUC_0-8_ was 22% and AUC_0-inf_ was 46% higher in women after 1-9 years after RYGB.	Only 1 post-bariatric occasion per patient.	[68]	Despite no clinically relevant differences in pharmacokinetics, resuming levonorgestrel after 1 year after RYGB in the absence of diarrhea may not be recommended routinely.
			20	1-8 years	No difference in levonorgestrel concentrations between patients who had undergone RYGB at least 1 year earlier and matched controls. There was a slightly positive correlation between AUC_0-inf_ and days after surgery, and a slightly negative correlation between AUC_0-inf_ and BMI. The longer the surgery dated back, the higher the AUC_0-inf_.	Only 1 post-bariatric occasion per patient.	[69]	
Immune suppressants	Tacrolimus	SG	1	2 weeks and 2 years	This patient underwent kidney transplantation 3 years before SG. Dose reductions were needed to achieve an adequate tacrolimus level. 2 weeks after an SG, the trough level was measured to be 18.8 ng/ml. The dose was reduced to 67%, with the tacrolimus level decreased to 5–7 ng/ml. After 24 months of follow-up, the tacrolimus level was 9–10 ng/ml. The dose was further lowered to 50%, resulting in adequate levels of 5–7 ng/ml.	Only published as abstract. Only trough levels were measured.	[70]	In patients who were already stable on immune suppressants: perform more intensive TDM in the first year after bariatric surgery. The AUC can increase over time, possibly requiring dose reductions.
			1	Multiple samples between 8–17 months	A heart transplantation patient had stable levels (goal 10–13 ng/ml) 8–12 months after SG. At 13 months after SG, the patient started to have variable levels leading to a moderate rejection despite adherence and no dose adjustments. Due to the high fluctuating tacrolimus levels, the dose required to achieve adequate concentrations ranged from 8–38 mg/day throughout the period of 8–17 months.	Only published as abstract.	[71]	
			12	9–12 months	In a study of patients with end-stage renal disease receiving immediate and extended-release formulations, the pre-SG and post-SG AUC_0-24_ were increased by 46% for immediate and 55% for extended-release formulation.	Only 1 post-bariatric occasion per patient	[72]	
		RYGB, SG	34	6, 12, 18, 24 and 30 months	No significant changes in dose adjustments after the surgery. Trend towards declining tacrolimus blood trough levels over time until 3 years after surgery, but the levels remained within therapeutic range. The number of patients with an adequate tacrolimus blood level tended to increase among RYGB patients, while it remained similar among SG patients.	Only trough levels were measured	[73]	

*AUC* = area under the curve, *AUC*_*0-inf*_ = area under the curve from 0 to infinity, *BD* = twice daily, *Cl* = clearance, *Cl/F* = apparent clearance, *Cmax* = maximal concentration, *F*= bioavailability, *Ka* = absorption constant, *N* = number of patients described, daily, *ref* = reference range, *RYGB* = Roux-en-Y gastric bypass, *SG* = gastric sleeve, *TDM* = therapeutic drug monitoring, *Tmax* = time to maximal concentration, *Vd* = volume of distribution, *Vd/F* = apparent volume of distribution

**Table 2:** Overview of the results from articles describing drugs with unchanged pharmacokinetics over time. Drugs are sorted by therapeutic group

Class	Drug	Type of surgery	N	Observation time points after surgery within the same patient	Changes in pharmacokinetics, effects or required dose	Remarks	References	Practical advice
No relevant changed pharmacokinetics over time								
Psychotropic drugs	Venlafaxine	RYGB	10	3-4 months	No changes in venlafaxine levels after single dose administration of extended-release venlafaxine		[74]	No dose adjustment needed when using extended-release formulation.
			5	1, 6 and 12 months	No changes in AUC_0-7_ and Cmax in venlafaxine-treated patients	Unspecified whether immediate release or extended-release formulation was used.	[3]	
		SG, RYGB	16	8 weeks, 6 and 12 months	Dose-adjusted concentrations were similar before and after RYGB or SG	Unspecified whether immediate release or extended release was used. Only trough levels were measured.	[30]	
Antibiotics	Ciprofloxacin	RYGB	17	1 and 6 months	AUC_0-14_ decreased by 4-20% within 6 months		[75]	No dose adjustment recommended
Cardial drugs	Rivaroxaban (only prophylactic dose of 10 mg reported)	RYGB SG	6 6	3 days and 6-8 months	Increase of median Tmax from 1.5 to 3 h. No change in Vd/F and AUC_0-24_ for a dose of 10 mg at 3 days and 6-8 months after surgery. Similar Cmax at 3 days and 6-8 months after surgery.	Caution needed for extrapolation of these results to higher doses, as absorption of rivaroxaban is non-linear and dependent on food intake.	[76, 77]	No dose adjustment needed for 10 mg dose. Due to non-linear absorption, the interpretation cannot be extrapolated to higher doses.
							[76, 77]	
Oral contraceptives	Desogestrel	RYGB	9	3 months and 1 year	No changes in AUC_0-24_ at 75 μg desogestrel (etonogestrel)		[78]	Despite no differences in pharmacokinetics, resuming desogestrel after 3 months after RYGB in the absence of diarrhea may not be recommended for all patients.
	Ethinylestradiol	RYGB	20	1-8 years	No change in ethinylestradiol concentrations between patients who had undergone RYGB at least 1 year earlier and matched controls.		[69]	Despite no differences in pharmacokinetics, resuming ethinylestradiol after 1 year after RYGB in the absence of diarrhea may not be recommended for all patients.
Other	Dextromethorphan	RYGB	10	5-8 weeks and 25-30 weeks	Cmax, Tmax, AUC_0-8_ were not significantly different at any time point. However, the standard deviation after surgery became smaller. There was a trend towards lower Cmax and AUC_0-8_.		[15]	No dose adjustment recommended.

*AUC* = area under the curve, *AUC*_*0-inf*_ = area under the curve from 0 to infinity, *Cmax* = maximal concentration, *F* = bioavailability, *N* = number of patients described, *RYGB* = Roux-en-Y gastric bypass, *SG* = gastric sleeve, *TDM* = therapeutic drug monitoring, *Tmax* = time to maximal concentration, *Vd* = volume of distribution, *Vd/F* = apparent volume of distribution

Based on the available data, various patterns were identified in altered pharmacokinetics in the short and long term. First, no pharmacokinetic changes after bariatric surgery were reported for 6 drugs: venlafaxine, ciprofloxacin, rivaroxaban (only prophylactic dose reported), desogestrel, ethinylestradiol and dextromethorphan. In addition, the pharmacokinetic changes over time were considered not to be clinically relevant for digoxin, omeprazole and levonorgestrel.

As a second group, we identified drugs of which the pharmacokinetics are significantly altered in both the short and the long term: lithium, tacrolimus, carbamazepine, duloxetine, midazolam, morphine, paracetamol, hydrocortisone, levothyroxine, rosuvastatin and metformin. Lithium levels can increase shortly after surgery [26–29, 79], which is attributed either to improved absorption of lithium or to a lower fluid intake resulting in a lower drug clearance. Consequently, severe lithium intoxications have been reported [26–28]. Most patients could be stabilized and discharged with a lower dose [26, 28, 79]. An a priori reduction of 30–70% in lithium dose is recommended in the first week after bariatric surgery, combined with monitoring of lithium levels (Therapeutic Drug Monitoring, TDM).

For other drugs in this group, such as tacrolimus and carbamazepine, intensive monitoring of drug levels shortly after surgery seems necessary. For duloxetine, reduced efficacy may be anticipated due to lower levels in both the short and the long term after bariatric surgery. In case of reduced efficacy, measuring duloxetine levels and increasing the dosage may be required. Studies on midazolam, morphine and paracetamol showed higher and earlier peak concentrations after surgery [16, 32, 64, 65]. This could potentially cause more adverse events at the time of maximal concentration (Tmax), while the clinical effects could fade off earlier. Hydrocortisone, levothyroxine, rosuvastatin, metformin showed various effects, as studies reported both increased and decreased levels over time. For these drugs, it is important to periodically monitor the effects through laboratory values and adjust the dose accordingly.

As a third class, we identified drugs that showed increased levels shortly after surgery and decreased values in the long term post-surgery. Among these drugs are pregabalin, lamotrigine, atorvastatin, simvastatin, warfarin, apixaban and amoxicillin. Additional clinical monitoring of effects and adverse events is recommended for pregabalin and lamotrigine. In case of epilepsy patients, measuring drug concentrations and adjusting doses accordingly can be helpful to prevent the occurrence of seizures. Atorvastatin, simvastatin and warfarin can be dosed according to their effects. Frequent LDL and INR measuring in the first few months after surgery can help to titrate the correct dose. Apixaban is a high-risk drug. Overexposure increases the risk of bleeding, while underexposure increases the risk of thromboembolic events. Hence, prescribers are advised to switch to drugs that can be monitored more easily, such as vitamin K antagonists.

Amoxicillin was shown to have initially increased exposure, followed by decreased exposure later on [39–41]. However, the results of these studies may have been influenced by differences in the formulation and dosages used, together with the known non-linear absorption of amoxicillin in higher doses [80]. Since the amoxicillin levels in these pharmacokinetics studies were all above the target minimum inhibitory value of 4 mg/L, no dose adjustments are advised for amoxicillin after bariatric surgery.

As a last category, the following drugs first showed lower drug levels, followed by higher drug concentrations that approached preoperative levels: sertraline, mirtazapine, phenytoin and probably citalopram/escitalopram. For these drugs, it seems important to monitor clinical effects in the short term. If altered effects are suspected, it is advisable to measure drug levels, i.e., to perform TDM, in addition to clinical monitoring. For epilepsy patients, it may be advisable to consider measuring phenytoin concentrations and adjusting doses accordingly.

For 7 drugs, data from less than 5 patients per drug was available (Table 3). Early after surgery, buprenorphine was absorbed faster, which normalized over time with the exposure decreasing by 40%. The plasma concentrations of tricyclic antidepressants seem to decrease over time. In contrast, the pharmacokinetics of gabapentin, topiramate and efavirenz did not alter over time.

**Table 3:** Reported changes in pharmacokinetics, effects or required dose of drugs after bariatric surgery that were described in less than 5 individual patients in total

Drug	Type of surgery	N (patients)	Observation time points after surgery within same patients	Changes in pharmacokinetics, effects or required dose	Remarks	Conclusions	Reference
Buprenorphine	SG	1	1 week, 1 month, 1 year	Buprenorphine sublingual: - at 1 week: similar AUC_0-24_ and almost doubling of Cmax compared to pre-surgery - at 1 month and 12 months: AUC_0-24_ decreased with 42–43%, while the Cmax returned to pre-surgery levels	Medication adherence not described	Cmax may be increased shortly after SG and may return to pre-surgery values after 1 month. The exposure may decrease after 1 month	[81]
Amitriptyline, nortriptyline, clomipramine	RYGB, SG	3	8 weeks, 6 months, 12 months	For the tricyclic antidepressants (TCAs) such as nortriptyline, amitriptyline and clomipramine, the drug levels seem to decrease over time, without any normalization to pre-surgery levels	Medication adherence was not described. Only trough levels were measured	Levels of TCAs may decrease over time and may require additional clinical monitoring. TDM may be performed	[30]
Gabapentin, topiramate	RYGB, SG	5	8 weeks, 6 and 12 months	In 2 gabapentin patients and 3 topiramate patients, no significant differences were found in dose-adjusted trough concentrations	Medication adherence was not described. Only trough levels were measured	There may be no differences in pharmacokinetics	[30]
Efavirenz	SG	1	3 and 6 months	After SG, pharmacokinetics was not altered in 1 patient compared to pre-surgery		The pharmacokinetics after SG may not be altered within 6 months	[82]

*AUC* = area under the curve, *Cmax* = maximal concentration, *RYGB* = Roux-en-Y gastric bypass, *SG* = gastric sleeve, *TCA* = tricyclic antidepressants, *TDM* = therapeutic drug monitoring

## Discussion

In this review, we describe results regarding pharmacokinetic changes over time for 39 drugs. Based on the findings of 56 included studies, we recommend that clinicians and pharmacists be aware of the pharmacokinetic changes that may occur over time. To support optimal therapy after bariatric surgery, more frequent clinical monitoring (i.e., drug counselling and outpatient follow-up) may be considered. Measuring specific drug levels in the first year after surgery could be helpful. Since some pharmacokinetic changes can still occur within the first year, further clinical monitoring may be required to ensure efficacy and safety in the longer term. For some drugs, no alterations in pharmacokinetics were identified. As such, in contrast to earlier reports in literature, there is no evidence to discourage the use of extended-release preparations (e.g., venlafaxine and morphine) after bariatric surgery [67, 74].

After bariatric surgery, patients may be more prone to adverse effects due to shorter gastric emptying time. Higher absorption rates and, consequently, higher and earlier peak concentrations (Cmax) could cause a quicker drug onset, which may be of relevance for sedatives or opioids such as midazolam and morphine. Thus, a change in the drug formulation, for example converting tablets into liquid, can have dramatic consequences, as was shown by a reported lithium intoxication [28]. Over time, the Tmax of most drugs becomes shorter, while the Cmax may vary over time: the concentrations either remain higher or return to preoperative values after several months.

Besides absorption-related differences due to intestinal adaptation, time-dependent pharmacokinetic changes can also be associated with reversal of obesity. Therefore, significant changes attributed to weight loss occur more gradually after bariatric surgery. One of these changes involves CYP3A activity, which is reduced in patients with obesity [83]. Assuming all other factors to remain similar after bariatric surgery, CYP3A activity normalizes over time, until it resembles the activity in patients without obesity [32]. However, due to changes in other physiological parameters, such as liver flow, this normalization may not necessarily translate into higher clearance of drugs that are hepatically metabolized by CYP3A [32].

Recent studies have shown that the pharmacokinetics of various contraceptives (i.e., desogestrel, ethinylestradiol and levonorgestrel) seem unaltered after RYGB [69, 78]. This finding is not in line with current advice that strongly discourages the use of certain drugs after bariatric surgery, such as oral contraceptives. This advice was based on a case series of 2 women who became pregnant after BPD despite the use of oral contraceptives [84]. A relevant culprit might have been the occurrence of chronic diarrhea in these women. Based on the recent studies, the absolute contraindication of any bariatric surgery for several oral contraceptives cannot be supported by pharmacokinetic data.

It should be noted that we did not a priori exclude case series, which can be considered a strength of our narrative review. In a systematic review, the extensive and very informative case reports of lithium intoxications would have been missed due to a low evidence level. Based on the scarce available evidence for most of the included drugs, we nevertheless tried to propose practical advice for clinicians. As for any review, the strength of this review is related to the strength of the reviewed papers. We realize that in the present review, the evidence was not graded and bias was not assessed in a systematic way, as was done by McLachlan [85]. Nor did we analyze the pooled data based on drug properties (e.g., lipophilicity, renal excretion, or involvement of CYP metabolism).

Clinicians may question how to dose patients who regain weight after bariatric surgery. A regain of weight of 10% or more occurs in 17.6% of the patients after bariatric surgery [86]. However, patients with weight regain were not specifically described in the included studies. That is why we could not assess whether patients with weight regain after bariatric surgery need different dosing advice over time.

Another question that remains to be answered is whether pharmacokinetics can be affected by differences in the practice of bariatric surgery. RYGB procedures differ between surgeons and clinics throughout the world in terms of the lengths of both the alimentary limb and the biliary limb, and therefore also in terms of the common channel. As far as we know, the effect of these differences on the pharmacokinetics of drugs have not been studied. However, as the current medication advice does not differ between RYGB and SG, we hypothesize that the differences in surgical practice may be of little clinical relevance.

Lastly, there is growing interest in microbiota changes after bariatric surgery [87, 88]. Obesity and weight loss seem to be associated with compositional changes in gut microbiota. More interestingly, the effect of weight loss on microbiota diversity seems to be time-dependent [87]. As microbiota can modulate both the absorption and the metabolism of drugs, they may constitute a missing link in the assessment of pharmacokinetic changes after bariatric surgery. To date, however, this is a highly understudied area.

This review highlights the importance of time-dependent dosing of drugs after bariatric surgery. In this complex matter, clinicians and pharmacists can collaborate in multidisciplinary teams to deliver the best care. This collaboration may involve additional or more intensive monitoring of clinical effects, performing TDM and adjusting doses. It should be acknowledged that information on the effects of bariatric surgery on the pharmacokinetics from 1 year and further on are very scarce. Thus, more research is needed to determine the optimal dosing at the time post-bariatric surgery, especially for drugs with a narrow therapeutic window.

## Conclusions

Pharmacokinetic changes after bariatric surgery can be time-dependent, leading to different changes in the short and long term after surgery. Due to the variability in the magnitude and directions of the changes in plasma concentrations over time, no general dosing advice can be provided. Besides, considering that the pharmacokinetic profiles of drugs may differ over time, it is recommended to monitor the clinical effects of certain drugs and, if needed, to measure drug levels and adjust drug doses accordingly. Consequently, close cooperation between clinicians and pharmacists is required to optimize drug doses for the intended clinical effect in post-bariatric patients.

### Supplementary Information

Below is the link to the electronic supplementary material.

Supplementary file1 (PDF 70 KB)
