# Supplementary material for: Impact of Bariatric Surgery in the Short and Long Term: A Need for Time-Dependent Dosing of Drugs
Source: Obes Surg. 2023 Aug 18;33(10):3266–302. doi: 10.1007/s11695-023-06770-5 (PMC10514130; doi:10.1007/s11695-023-06770-5)
Supplement: Supplementary file 1 — Supplementary file1 (PDF 70 KB) [file 11695_2023_6770_MOESM1_ESM.pdf]

Supplementary materials

Search strategies used:

PubMed

1. bariatric* [TiAb] OR (Roux-en-Y gastric bypass) OR (Roux-en-Y*) OR (gastric sleeve) OR (sleeve gastrect*) OR (gastric banding) OR (bariatrics[MeSH Terms]) or (gastric bypass[MeSH Terms]) or (sleeve gastrectomy[MeSH Terms])
2. (drug concentration) OR (drug level) OR pharmacokinetics OR “pharmacokinetics” [MeSH Terms]
3. (time after surgery) OR change* OR “time” [MeSH Terms] OR “long-term” OR “short-term”
4. 1 AND 2 AND 3

Embase

1. ‘bariatric surgery’/exp OR ‘bariatric surgery’ OR bariatric* OR ‘sleeve gastrectomy’/exp OR ‘sleeve gastrectomy’ OR ‘sleeve gastrect*’ OR ‘roux-en-y gastric bypass’/exp OR ‘roux-en-y gastric bypass’ OR ‘roux-en-y*’ OR ‘gastric bypass’/exp OR ‘gastric bypass’ OR ‘gastric band*’
2. ‘drug concentration’ OR ‘drug level’ OR pharmacokinetics OR ‘pharmacokinetics’/exp
3. time AND after AND surgery OR change* OR alter* OR time OR ‘long term’ OR ‘short term’
4. 1 AND 2 AND 3
